# Ethnographic process evaluation of a quality improvement project to improve transitions of care for older people

**DOI:** 10.1136/bmjopen-2015-010988

**Published:** 2016-08-04

**Authors:** Elizabeth Sutton, Mary Dixon-Woods, Carolyn Tarrant

**Affiliations:** 1 Department of Health Sciences, Social Science Applied to Healthcare Improvement Research (SAPPHIRE) Group, University of Leicester, Leicester, UK; 2 Cambridge Centre for Health Services Research, Department of Public Health and Primary Care, University of Cambridge, Cambridge, UK

**Keywords:** GERIATRIC MEDICINE, QUALITATIVE RESEARCH, transitions of care, care homes, readmissions

## Abstract

**Objectives:**

Quality improvement projects to address transitions of care across care boundaries are increasingly common but meet with mixed success for reasons that are poorly understood. We aimed to characterise challenges in a project to improve transitions for older people between hospital and care homes.

**Design:**

Independent process evaluation, using ethnographic observations and interviews, of a quality improvement project.

**Setting and participants:**

An English hospital and two residential care homes for older people.

**Data:**

32 hours of non-participant observations and 12 semistructured interviews with project members, hospital and care home staff.

**Results:**

A hospital-based improvement team sought to reduce unplanned readmissions from residential care homes using interventions including a community-based geriatric team that could be accessed directly by care homes and a communication tool intended to facilitate transfer of information between homes and hospital. Only very modest (if any) impacts of these interventions on readmission rates could be detected. The process evaluation identified multiple challenges in implementing interventions and securing improvement. Many of these arose because of lack of consensus on the nature of the problem and the proper solutions: while the hospital team was keen to reduce readmissions and saw the problems as lying in poor communication and lack of community-based support for care homes, the care home staff had different priorities. Care home staff were unconvinced that the improvement interventions were aligned with their needs or addressed their concerns, resulting in compromised implementation.

**Conclusions:**

Process evaluations have a valuable role in quality improvement. Our study suggests that a key task for quality improvement projects aimed at transitions of care is that of developing a shared view of the problem to be addressed. A more participatory approach could help to surface assumptions, interpretations and interests and could facilitate the coproduction of solutions. This finding is likely to have broader applicability.

**Strengths and limitations of this study**

- Strengths of this study include its use of a concurrent qualitative process evaluation to study an improvement project and the independence of the evaluation team from the quality improvement team.
- The conclusion that interorganisational quality improvement should be recast as a participatory activity that starts with problem definition has applicability beyond the specific context of the study.
- Owing to the nature of the quality improvement project, this study was limited to one hospital and its associated catchment of care homes.
- The study team did not have direct access to the raw data on hospital readmissions used by the quality improvement team.

## Introduction

Though transitions of care between organisations are increasingly attracting intense interest, particularly where financial penalties have been directed towards hospital readmissions,^1^ ^2^ suboptimal transitional care remains a stubborn feature of many health systems.^3^ ^4^ An extensive literature has repeatedly described serious problems of continuity and coordination relating to the care of older people in particular, linked to adverse events, lower patient satisfaction and high rehospitalisation rates.^5^^–^8 Older people living in residential care (including nursing homes) appear to be especially vulnerable.^9^

Many of the reasons for poor transitions of care for older people, including intersectoral conflict, poor handover of information and weaknesses in operational processes, are increasingly well understood.^10^ ^11^ In response, academic research and reports have called for leadership, partnership and quality improvement to enable better working between the sectors caring for older people.^4^ ^12^ ^13^ However, turning these aspirations into reality has not been straightforward:^14^ quality improvement efforts, in this area as in others, do not always deliver the desired success.^15–17^ Though some of the generic challenges of quality improvement are becoming clear,^18^ the reasons why quality improvement projects to address the problems associated with transitions of care produce sometimes disappointing or weakly sustained results have remained elusive. An enduring frustration is many quality improvement projects, in this area as in others, are poorly evaluated, often relying on self-assessment by the team leading the project,^16^ and often lack concurrent process evaluation that can investigate influences on implementation.^19^

Better understanding of why quality improvement is so hard in the context of transitions of care for older people is much needed. An important opportunity to address this need arose in the context of an independent evaluation that we conducted of projects participating in a healthcare service improvement programme. This programme, known as the Health Foundation's Safer Clinical Systems programme, trained and supported clinical teams in the English National Health Service (NHS) to diagnose hazards along their clinical pathways using techniques adapted from high-risk industries, and then to use quality improvement methods to address the problems identified.^20^ One of the projects was led by a hospital-based team that sought to improve care of older people in transitions between hospital and residential care settings (including nursing homes) during periods of acute illness. In this paper, we report an independent process evaluation that sought to characterise the challenges experienced by this quality improvement project, focusing specifically on those that arose from its cross-boundary nature.

## Methods

We conducted an independent ethnographic process evaluation of a Safer Clinical Systems project led by a team based at a hospital that we anonymised as Oaktree.

Thirty-two hours of non-participant observations were conducted over the course of the project by a non-clinical researcher (ES), who was a member of the independent evaluation team for the Safer Clinical Systems programme. Observations were recorded in the form of field notes across Oaktree's Emergency Department, two care homes (one residential, one that provided residential care and nursing care) and the local Clinical Commissioning Group (the NHS organisation responsible for local commissioning of secondary care and community health services). The care homes had been selected for inclusion in the quality improvement project by the improvement team because of their high numbers of readmissions. Observations in the care homes took place in the homes' day care rooms and involved discussions with staff, including care assistants and care home managers/owners, mostly during the day. Observations were also conducted at two events known as Care Home Forums, which were open to general practitioners, care home managers and staff, community and hospice nurses, and social workers. Relevant project documents, including meeting minutes, progress reports and other outputs of the project team, were also collected and included in the analysis.

We conducted 12 interviews with individuals involved with the project: 6 with project and hospital-based staff (who were mainly, but not exclusively, based in the emergency department) and 6 in the community (including care home managers and front-line care home staff). Interviews were semistructured and were mostly conducted face to face, with some conducted by telephone for the convenience of the interviewee. Seven face-to-face interviews were recorded as field notes. Signed consent was obtained prior to interview, and those that were recorded were transcribed verbatim. All interviews were anonymised.

Data on readmissions and other measures were collected by the hospital improvement project team. The raw data were not available to the independent evaluators, but the project team produced summary charts that we reviewed.

Data analysis was based on the constant comparative method,^21^ assisted by NVivo software. Initial open coding was applied to the transcripts and field notes, and then used as the basis of provisional thematic categories. Iteratively comparing these categories against the data and each other, and using sensitising constructs identified from the literature, allowed the generation of higher order themes and concepts used to categorise the data.

## Results

### Activities undertaken by the improvement team

Applying the techniques they had learnt as part of the Safer Clinical Systems programme, the Oaktree quality improvement project team sought to diagnose the reasons underlying unplanned readmissions, and then developed and implemented interventions to address the problems they had identified. The team found that the highest category of readmissions was associated with the care home sector, and concluded that there was a need for better access to medical, nursing and therapy services in the community to support patients and reduce readmissions.

Communication between care homes and hospital was identified as a particular problem. The diagnostic work found that patients often arrived from care homes at hospital by ambulance with no accompanying carer, and with very little medical information. These problems were compounded when patients themselves were unable to communicate (eg, due to dementia or delirium). As a result, clinicians in the emergency department were often unclear about what they could do to help, resulting in patients being returned to residential care without significant medical intervention.

One 90-plus year old was sent the emergency department by care home with no carer and was confused. He had a minor injury to his arm. His daughter had phoned and explained he was suffering from dementia. Doctor said that there was nothing medically he could do for him and did not really understand what he was doing there. *(Field notes)*

Communication of information from the hospital to care homes at discharge was also found to be problematic, in part because discharge summaries went to patients' general practitioners rather than to the care homes.

The early diagnostic phase, we realised that our own discharge notification was inadequate, and in terms of the information we were giving, was very health-to-health. We also knew that the care providers at times weren't allowed to see it, so they couldn't read what was in it and as a result of obviously then had no inkling of what to do. (*Interview, Oaktree project team*)

The diagnostic work further suggested that a major barrier was lack of clarity about who would assume the responsibility for ensuring that an adequate plan was in place to support patients post discharge and for communicating about this plan with the care homes.

We assumed that the reason for readmission was purely due to a poor handover, but we were able to establish that a significant reason was the lack of an adequate team [in the community] to receive the handover*. (Document—Oaktree team interim progress report)*

The quality improvement interventions selected by the Oaktree team to address these problems included the introduction of a community geriatric service with a dedicated geriatrician and a community matron (senior nurse) and a 24-hour telephone support service. The service could receive handovers for patients discharged back to care homes, and could be accessed directly by care homes for advice. The team also introduced a summary information form, intended to accompany the patient between care home and hospital, to improve communication and information transfer.

The Oaktree team monitored readmission rates between September 2011 and October 2013, covering a baseline period and the introduction of the community geriatrics team in mid-2012. Summaries of these data were made available to the evaluation team and indicated that the impact of the project appeared to have been (at best) very modest: it was difficult to detect, from the data collected, evidence of any major shift in readmission rates.

### Challenges in implementing interventions and securing improvement

The process evaluation identified many challenges in implementing interventions and securing improvement. Although the two interventions selected by the project team were carefully designed to address the problems identified through the diagnostic work, they were not straightforward to implement. One reason for this was located in historically poor relationships characterised by mistrust and suspicion between the hospital and local care homes. Members of the Oaktree project team worked hard to improve these relationships and foster goodwill by, for example, attending local care home forums and undertaking voluntary work at care homes. The team also sought to involve care homes in the design and piloting of the summary information tool. These efforts did meet with some success, but the process of developing relationships was slow.

So it's actually taken quite a long time to establish the relationship with the residential nursing home and GPs within the area. And the good thing is we now have got some good relationships, but the bad thing is it's taken quite a lot longer to develop those than we thought it might. (Interview, project team member)

Many of the difficulties lay in broader challenges of problem definition. The Oaktree team saw the problems as lying primarily in communication between care homes and the hospital, and in behaviours of care homes linked to the inadequacies of community-based support for the homes. However, observations and interviews with care home staff made it clear that their perspectives on the causes of the problems—and consequently how they should be fixed—were different from those of the quality improvement team. Even though the Oaktree project team had emphasised the hospital's role, care home staff reported that they felt that problem of care transitions had been defined largely from the point of view of the Oaktree project team, and reflected a preoccupation with hospital concerns—specifically readmissions. Yet, as far as care home staff were concerned, much of the responsibility for readmissions was located with the hospital, not with them.

Care home staff suggested patients were sometimes discharged from hospital even when, from the perspective of care homes, it was neither appropriate nor safe to do so. Hospital priorities, they suggested, were heavily skewed towards efforts to free up beds and a perceived ‘patch them up and send them out’ mentality.

Her emphasis was very much “I don't understand, we send patients into hospital and they don't do anything for them and they just send them out. So there's no follow up, there's no aftercare” and she was adamant that this was because a lot of residents have got dementia and a lot of them are seen by hospital staff as “not worthy of treatment.” *(Field notes)*

Care home staff further suggested that hospital definitions of ‘good’ care for frail older people differed significantly from the definitions and understanding of good care that underpinned care home practice, resulting in what care home staff saw as unsafe or inappropriate decisions about discharge. Mistrust and conflict then followed.

Care home managers seemed concerned that people were coming back from the hospital on the same day [as they went in] and then they had to send them back to the hospital within 48 hrs. *(Field notes)*

Accordingly, care home staff, rather than seeing their own practices as contributing to readmissions, were critical of the hospital's approach to managing older patients and its perceived failure to ensure consistently safe discharge. Poor discharges, they argued, transferred risk to the care homes. Care home staff suggested, for example, that hospitals often discharged residents back to care homes at the weekend without necessary medication or equipment, leaving the home vulnerable: they felt they were *‘*‘*stuffed’ [open to being blamed] when that happened’* (Interview, care home staff).

As care homes and the hospital did not share a common understanding of the nature of the problem and the reasons for it, care home staff did not fully accept that the interventions introduced by the project team were necessarily the optimal ones for addressing the problems they perceived. For example, care home staff criticised one of the quality improvement interventions—a brief discharge summary on the back of the summary information form to be completed by hospital staff—suggesting that it was inadequate for meeting standards of safe discharge. Care home staff thus perceived that the hospital was not genuinely investing effort into improving its discharge processes.

A lot of the care homes were quite hostile […] and the reason they were quite hostile was they've had some bad experiences, they felt that their patients were not getting good care from the hospital, they were just sending them out at all hours of the night, they weren't talking to them about what the problem was, they weren't sending the right forms back in, they don't know what the patient had come back out with, what the issues were. *(Field notes)*

Perhaps most challenging of all, care home staff felt that the complexities of their decision-making when their residents were acutely ill had not been fully recognised by the hospital project team, meaning that the effectiveness of the other major quality improvement intervention—the community geriatric team—was compromised. A key issue was that care homes were anxious about being found legally liable for failing to act on concerns about their residents' health. Care home staff were therefore often reluctant to do anything other than telephone for an ambulance in the event of a resident's falling or becoming ill, for fear of being accused of neglecting their duty of care. If using the new geriatric team to avoid readmission was likely to introduce delay in a patient getting medical care, they preferred to send patients straight into hospital rather than risk patient deterioration or death at the care home and subsequent exposure to litigation or accusation.

The care home gave an example of a lady who they admitted back to the home [after hospitalization] but then they were unable to get her blood sugars back up. I asked if she had considered phoning the community matron but she stressed that she wasn't prepared to wait. She needed an immediate response. *(Field notes)*

There's a big issue around training about when you should call for an ambulance and also responsibility because a lot of the younger carers won't necessarily have the skills. There is also concern about whose responsibility it is if something goes wrong and they're not sent off to hospital there is a fear, a deep-rooted fear, that the patients or family will sue the home. That is a big issue for why [the care home] constantly pick up the phone and get them sent in. *(Field notes)*

Patients at the end of life were seen as particularly at risk of emergency readmissions, yet care home staff reported that their decisions and behaviours about managing patients who were dying were extremely difficult, being heavily influenced by perceptions of accountability and liability for their actions, and shaped by their relationships with the patient and their families. Addressing the issue of avoiding readmissions for this patient group could not be solved by simple interventions; instead, they required attention to more difficult issues of communication with patients and relatives about end-of-life care and preferences about the best place for delivery of care.

The care homes are frightened if they don't send people at end-of-life into hospital there'll be questions asked. (*Field notes)*

One of things is trying to get care homes to get them to sit and down and talk to the patients, or if not the patients if they're not capable then the patient's relatives, about end-of-life planning—and this care home manager was absolutely adamant there was no way she was going to do that. “It's uncomfortable” “it's not something that we should be doing.” *(Field notes)*

## Discussion

Progress on improving transitional care for older people has been frustratingly slow, and quality improvement is frequently advocated as a solution.^12^ This study characterises the challenges of a quality improvement project that sought to improve transitions of acutely unwell older people between hospitals and residential care, but produced only modest (if any) impacts on readmission rates. The ethnographic process evaluation uncovered tensions in the goals, priorities and values of the hospital and local care homes that frustrated improvement work across organisational boundaries. Many of these challenges arose because of lack of consensus on the nature of the problem and the proper solutions.^18^ These findings suggest that a key task for quality improvement projects that focus on improving transitions of care for older people is that of developing shared definitions of problems and coproducing solutions.

As earlier work has shown, improving care across organisational boundaries, particularly when it involves multiple partners and funding streams across secondary and community care,^22^ ^23^ is likely to require efforts to develop trust, good relationships and effective communication between organisations.^24^ For instance, providing geriatric or acute care nursing experience input into care homes may reduce demand on local emergency departments and reduce readmissions,^25^ ^26^ but the quality of relationships built with the nursing home managers and healthcare staff is likely to be essential.

This study adds to this literature in drawing attention to the importance of problem definition as a focus for quality improvement work. Work in other fields has shown that who gets to define the problem plays a crucial role in the ensuing actions, in shaping the solutions to that problem and in governing organisational practice.^27^ The ‘problem’ in this improvement project was defined by the hospital project team as unscheduled readmissions, founded in poor communication between hospital and care homes and lack of access to expertise in managing acute illness in older people. These definitions of the problem were not fully shared by care home staff, however, who instead pointed to their perceptions of the poor practices of the hospital. Rather than prioritising the reduction of readmissions sought by the hospital, care homes were attentive to the possibility of litigation and accusation. The proposed solutions thus failed to align with what they saw as necessary. The underestimation of the complexity of care transitions and their tractability to intervention that was a feature of this project is a common finding in other research in transitional care,^28^ ^29^ where there has been a history of imposing top-down solutions. The value of our study is in demonstrating that quality improvement efforts in complex, cross-boundary settings are particularly likely to benefit from a participatory approach to problem definition and selection of interventions.

A commitment to coproducing^30^ the nature of the problem and the solutions may help to avoid some of the pitfalls that can derail well-meaning interventions that are designed and implemented with a one-sided perspective. A more participatory, coproduced approach could help to surface assumptions, interpretations and interests, allowing for differences to be made explicit and for their implications to be anticipated. Approaches such as experience-based codesign may be especially well placed to facilitate such work,^31^ since they see identification of the key actors (including, in this instance, patients/care home residents and their families as well as staff in different locations) as an important first step that is then followed by active engagement with these actors over the course of the project. Such an approach would emphasise the need for reconciliation and alignment of perspectives, including, where necessary, mechanisms for conflict resolution.

This study's strength lies in its explication of different perspectives that revealed why it may be so difficult to make improvements across sectors and in its use of ethnographic methods that allowed in-depth insight into the project as it unfolded in multiple locations. The independence of the study team from the quality improvement team helped to produce an impartial account of these challenges, which might otherwise have remained invisible or been uncomfortable to identify.^32^ However, it also has some limitations. Given the nature of the quality improvement project, our study was necessarily confined to one hospital and its associated catchment of care homes. It did not investigate in detail contextual features of the organisations involved (eg, workforce turnover, ratio of qualified to unqualified staff). It also did not access the views of older people and their families, and this should be a focus of future work. A further limitation is that, while it was possible to review summary charts on readmissions generated by the project team, direct access to the raw quantitative data on admissions was not available.

## Conclusions

Quality improvement aimed at interorganisational transitions remains challenging, and process evaluations have a valuable role in providing insights into the nature of these challenges. Our study suggests that a key task for intersectoral, cross-boundary quality improvement projects care is that of developing a shared view of the problem to be addressed. A participatory approach could help to surface assumptions, interpretations and interests that can, unless aligned, may thwart cooperation and undermine progress. Quality improvement interventions are likely to benefit from being coproduced by all those involved. These findings are likely to have broader applicability in quality improvement efforts across organisational boundaries.
